# Introduction of Voluntary Medical Male Circumcision for HIV Prevention in Tanah Papua, Indonesia

**DOI:** 10.1007/s10461-025-04704-z

**Published:** 2025-04-22

**Authors:** Robert C. Bailey, Ignatius Praptoraharjo, Nidia Muryani, Daniel Ardian Soeselo, Shanta Ghosh, Judith A. Levy

**Affiliations:** 1 School of Public Health, University of Illinois Chicago, Chicago, IL USA; 2 Health Policy and Social Innovation, University Centre of Excellence-HIV AIDS Research Center, Atma Jaya Catholic University of Indonesia, Jakarta, Indonesia; 3 Department of Surgery, School of Medical and Health Sciences of Atma Jaya Catholic University of Indonesia, Jakarta, Indonesia

**Keywords:** Medical male circumcision, Human immunodeficiency virus, Papua Indonesia, Community Participatory approach, Safety, Acceptability

## Abstract

Tanah Papua, comprising Papua and West Papua *(Papua Barat)*, includes only 1.5% of Indonesia’s total population but accounts for over 15% of the country’s new human immunodeficiency virus (HIV) cases. Overall, adult HIV prevalence in Indonesia in 2018 was 0.26%; in Papua it was nearly ten times higher at 2.3%, and almost all new infections occurred through heterosexual transmission. Being a predominantly Muslim country, male circumcision (MC) is nearly universal in Indonesia except in Papua where MC is little practiced. The Indonesian government has turned attention to World Health Organization/UNAIDS 2007 recommendations to offer voluntary medical male circumcision (VMMC) in Tanah Papua as part of a comprehensive package of HIV services. Currently, there are no functioning VMMC programs designed specifically for Papua or with input by Papuans. Using a community-participatory approach, we developed and pilot-tested the Papua Indigenous Model (PIM) of VMMC for acceptability, feasibility, and safety as part of a comprehensive HIV prevention strategy to reduce HIV sexual transmission in Papua. The model of VMMC was developed based on numerous meetings with government officials, health providers, teachers, students, parents, and community leaders. In total, 88 adults and 31 adolescents provided input during focused group discussions. Thirty-two meetings were held with approximately 1050 community members. Staff at three community health facilities were trained in safe VMMC services according to WHO guidelines. While all males ages 15 and above were eligible for VMMC, recruitment by trained peer outreach workers focused especially on Papuan ethnic males ages 15–19 years. Based on other VMMC programs and our consultations with community members, we expected to screen 400 potential participants, but ultimately only 104 participants volunteered to be screened at the three facilities. Of the 104, 94 participants were eligible and accepted circumcision. The average pain score reported by participants was low: 3.4 at 30 min post-circumcision. Two participants (2.1%) experienced a moderate adverse event (AE); no severe AE occurred. Upon follow-up, 98% said that they were somewhat or very satisfied with the procedure; 98% agreed that *“If I had a son*,* I would get him circumcised;”* and all but three participants reported that if they had it to do again, they would get circumcised. Despite extensive consultation with local communities, VMMC uptake was lower than anticipated, while levels of satisfaction among those circumcised were high. Even with substantial community input into its promotion, achieving a successful scale-up of VMMC in Papua will be highly challenging, requiring significant support from local, national, and international stakeholders. Nevertheless, it should be made available, integrated with the numerous other evidence-based HIV prevention measures.

## Introduction

Composed of over 6000 inhabited islands with approximately 270 million people, Indonesia has multiple intersecting human immunodeficiency virus (HIV) epidemics affecting the archipelago’s different sub-populations and locations disproportionately [1]. Tanah Papua, composed of both Papua and West Papua *(Papua Barat)*, with only 1.5% of the country’s total population, accounted for over 15% of Indonesia’s new HIV cases in 2011 [2]. In Indonesia overall, adult HIV prevalence in 2018 was 0.26%, whereas in Papua, estimates were nearly ten times higher at 2.3% in 2020, and trends in testing results suggest that the epidemic is increasing in scale. The province has the highest prevalence among major administrative areas in the Asia-Pacific region and prevalence is higher than the neighboring country of Papua New Guinea [3, 4]. While in other regions of Indonesia the HIV epidemic is predominantly among men who have sex with men and people who inject drugs, in Papua almost all new infections occur through heterosexual transmission [1, 3, 4]. Further, as a predominantly Muslim country, male circumcision is nearly universal in Indonesia except in Papua where MC is traditionally practiced primarily by migrants from other regions of the country and by a few small, isolated ethnic groups, often with only partial removal of the foreskin [1–3, 5].

HIV prevention efforts based on abstinence or safer sex have shown only modest success in Papua. Pre-exposure prophylaxis (PreP) has been approved solely on a pilot basis in 22 Districts in Indonesia, none in Papua [6]. As a result, the Indonesian government has turned attention to the World Health Organization/The Joint United Nations Programme on HIV/AIDS (WHO/UNAIDS) 2007 recommendations to offer voluntary medical male circumcision (VMMC) as part of a comprehensive package of HIV services for Tanah Papua. A one-time intervention, VMMC provides men with approximately 60% protection against HIV [7–9]. A challenge for Indonesia in adopting this strategy is the lack of research to guide and ensure the effort’s success. Also, Papuans tend to mistrust government intervention programs introduced to Papua without input or buy-in from Papuan leaders or without regard for its indigenous cultural traditions, religious beliefs, and special environmental challenges [10]. Currently, no functioning VMMC programs exist designed specifically for Papua or with input by Papuans.

Using a community-participatory approach, we developed and pilot-tested the Papua Indigenous Model (PIM) of VMMC for acceptability, feasibility, and safety as part of a comprehensive HIV prevention strategy to reduce HIV sexual transmission in Papua.

## Methods

### Community Engagement

Preparations for the study began in Papua in August 2022. To engage Papuan leaders and community members in exploring acceptability, barriers, and facilitators for introducing a culturally appropriate, comprehensive VMMC intervention, we met with members of the Ministry of Health of the Republic of Indonesia and conducted a series of meetings with the heads of the Ministry of Health and the Ministry of Education in Jayapura and in Nabire. The purpose of these initial meetings was to inform the national and provincial authorities about the aims of the study, gain their suggestions regarding access to local leaders and authorities, and obtain formal letters of permission to conduct the study. To enhance community engagement in the model’s development, 15 focused group discussions (FGD) were conducted: 7 in Jayapura and 8 in Nabire districts. There were 119 participants: 88 adults over age 17 and 31 adolescents ages 15–17 years. Concurrently, we conducted 17 in-depth interviews with 13 males and 4 females to identify facilitators and barriers to implementing VMMC within a Papuan context. Based on the information collected during the FGDs and interviews, we subsequently conducted 32 meetings with community groups: 21 in schools and 11 in social halls, churches, or community settings. A total of approximately 1050 individuals participated in the meetings, providing opinions and suggestions regarding the design and best implementation practices for the PIM. Based on the results of those meetings with community members, elders and village leaders, students and teachers, we developed the PIM and the plan for introducing VMMC in community health facilities (*puskesmas*) in Papua’s Nabire District. In consultation with local stakeholders, we chose Nabire District because of its high proportion of indigenous Papuans and annual HIV rates that are among the highest in all of Tanah Papua.

### Ensuring VMMC Capacity

To assess the feasibility of implementing a VMMC program within the context of Papua’s community health system and to evaluate the system’s capacity to meet international criteria for safe, comprehensive VMMC services, we assessed the resources and provider training available to meet these standards in four selected *puskesmas* in Nabire district. In consultation with health providers and community members, three of these *puskesmas* were chosen to begin training medical providers and staff for delivering VMMC services. Materials, supplies, and equipment were purchased to bring each facility up to WHO standards for the provision of safe VMMC. The purchases included autoclaves, air conditioners, surgical lamps, and expendable clinical supplies. We then conducted a continuing medical education (CME) for all providers in the three facilities describing the aims of the project and instruction on how safe VMMC is performed, including counseling and HIV testing, screening participants for medical and age eligibility criteria, performing the circumcision, post-op care and behavioral counseling – all adapted from the WHO/JHIPIEGO Manual for Male Circumcision [11].

A total of eight providers-three medical doctors and five nurse-assistants—were trained to perform the circumcision procedure using the dorsal slit method [11] by Daniel Ardian Soeselo, MD, Surgeon, from the Department of Surgery, School of Medicine and Health Sciences of Atma Jaya Catholic University. Staff at each facility were also trained in sterilization protocols, data entry, and data management.

### VMMC Recruitment Procedures and Eligibility

A meeting was held on October 20, 2022, with 25 key stakeholders (heads of health facilities, heads of the District hospital, and heads of schools) to finalize the plan for introducing the indigenous-based model of VMMC. As agreed, active recruitment focused on adolescent males ages 15–19 years in primary and secondary schools; however, males of any age from 15 years were eligible. Peer research assistants gave talks at schools and social gatherings, explaining the availability of the services and the risks and benefits of VMMC. Fliers were distributed, and interested participants were given the opportunity to volunteer for VMMC.

To be eligible for VMMC, males had to be uncircumcised, age 15 years or above, have no signs or symptoms of a sexually transmitted infection (STI) nor any penile abnormality (e.g., hypospadias, phimosis, or steroid injections) contra-indicating circumcision, nor any history of a bleeding disorder. According to the Indonesian Ministry of Health, anyone under the age of 18 years is considered a minor. Thus male volunteers between ages 15 and 17 years were required to provide signed informed assent, with signed informed consent provided by at least one parent or guardian. Men older than 17 years had to provide signed informed consent. Anyone under the influence of alcohol or drugs was excluded.

Participants coming to the facility to volunteer for VMMC were counseled about the risks and benefits of participating in the study and asked to agree to be tested for HIV. Those who consented were asked their age, education, residence, sexual history, and beliefs about male circumcision and health. They were counseled and tested for HIV by a trained, certified nurse or doctor. Those who tested seropositive were referred within the facility to a program offering social and medical services and anti-retroviral therapy for people living with HiV. They were given the option of being circumcised, but none accepted. Volunteers who tested seronegative were referred to the surgical team, asked for a medical history, and examined for signs of an active STI or other conditions that might make them ineligible for circumcision.

### Post VMMC Procedures

VMMC procedures began March 31, 2023 and ended August 28, 2023. Participants remained in the *puskesmas* for at least one hour for observation post-procedure. If fully recovered and medically stable, they were given a soda and biscuits, directions on wound care, and information on how to access a 24-h hotline should they have any questions or concerns. Seven days post-circumcision, they returned, the wound was inspected, and they were asked a series of questions about their experience with pain, level of satisfaction, and any sexual behavior since circumcision. Participants returned six weeks later post-circumcision to assess wound healing, level of satisfaction, sexual activity, and whether they had experienced any harassment or discrimination due to being circumcised. Any adverse events were addressed by the clinicians and categorized as mild, moderate or severe. Safety was measured as the number of moderate or severe adverse events as a proportion of the total number of circumcisions performed.

Originally, we had planned to assess acceptability by recording all participants between ages 15–19 years who had attended information and recruitment sessions at schools or social settings. However, this proved infeasible since, first, many of those attending sessions came and left and were not recorded as present, and, second, many males were exposed to VMMC messages outside these settings in public areas and by word of mouth, making recording their exposures to messaging impossible. Consequently, we assessed acceptability as the number of boys who volunteered for VMMC compared to the number we expected (400) prior to the onset of the five months of implementation.

### Data Management and Analyses

Data were recorded on hardcopy forms and entered by a designated data clerk at each *puskesmas* into Kobo Toolbox, an open-source data collection platform. All KoboToolbox accounts (https://support.kobotoolbox.org/is_my_data_safe.html*)* are password-protected, and all database content is encrypted at rest (disk-level encryption) and sent encrypted to the designated server for analysis. Data checks were performed daily by a trained research assistant who followed up to see that any missing data or errors were addressed within days of data collection. Data were downloaded for analyses using the R statistical computing and graphics system (R Core Team [2023] R: https://www.R-project.org).

Results were stratified by participants who were indigenous Papuan versus non-Papuans, since most non-Papuans circumcise routinely and may have characteristics, behaviors or views different from Papuans. Mean ± standard deviation (SD) or median ± inter-quartile range (IQR) and proportions were calculated for continuous and categorical variables, respectively. A t-test or Wilcoxon rank-sum test assessed the difference in continuous variables by location (non-Papua vs. Papua), and a Chi-squared test or Fisher’s exact test was used to assess a difference in categorical variables. A two-sided p-value of ≤ 0.05 was considered statistically significant.

The study was approved by the University of Illinois Chicago Institutional Review Board (IRB) #1, by the Atma Jaya Catholic University IRB, and by the National and Political Unity Agency of Papua Province.

## Results

Implementation of VMMC was initially delayed due to COVID-19 travel restrictions in Indonesia, and there were several subsequent delays due to political turmoil, physical violence, and religious holidays. VMMC procedures began March 31, 2023 and ended August 28, 2023. A total of 104 volunteer participants were screened at the three health facilities. Acceptability was assessed as the number of boys (104) who volunteered for VMMC compared to the number originally expected (400). Of the 104 who volunteered, four were not enrolled in the study: two were underage, one did not get parental consent, and one opted out of participation. Of the remaining 100, three were found to be medically ineligible for circumcision due to steroid injections to enlarge their penis and foreskin, two tested HIV positive and were offered but declined circumcision and were referred to counselors for discussion of ART initiation, and one additional person who was eligible for circumcision declined it. In total, 94 participants were circumcised. While a follow-up visit three days post-circumcsion was not part of the original protocol, staff at the health facilities decided to ask participants to return after three days for a check-up and change in bandages. Eighty-four (89%) participants returned for the three-day follow-up visit, and 87 (93%) returned for the scheduled 7-day follow-up visit. Ninety (96%) participants were either seen at the *puskesmas* for their 6-week post-op visit or interviewed by cell phone.

Although we accepted any men over age 14 who sought circumcision, we specially targeted 15–19 year-olds. Despite this, just 52 participants (55%) were in the age category 15–19 years; the remainder 42 (45%) were 20–44 years. The median age of participants was 19 years (Interquartile Range (IQR) 17, 22). We focused our recruitment efforts in schools, and 70% of participants were current students. We also aimed to recruit primarily indigenous Papuan males; however, just 58 (62%) of those circumcised identified as indigenous Papuan. The remainder were immigrants from other Indonesian islands, some of which are not predominately Muslim or practice male circumcision.

The median time for the circumcision procedure, measured from the time of first incision to completion of bandaging, was 40 min (IQR: 34, 45). There were no severe adverse events (AE). There were two moderate AE. In one case, balloon swelling occurred between stitches within two hours after the procedure. Pressure was applied and the wound was rebandaged. On Day 3, there was some bleeding. The wound was irrigated with saline solution, pressure was applied and the wound was rebandaged. When the patient returned on Day 7, there was no bleeding and the wound was healing normally. In the other case, bleeding after surgery could not be stanched with pressure. The participant was taken to the Outpatient Emergency Room at the District Hospital. It was determined that four small vessels were bleeding. Some stitches were removed, the bleeders tied, the wound closed with six stitches, the wound rebandaged, and the participant was transported home with instructions for wound care. When he returned on Day 3, there were no signs of bleeding, and the wound was progressing normally, similarly on Day 7.

Before the surgical procedure, when asked to give the main reason that they wanted to be circumcised, 76% of participants responded about hygiene, and 12% responded about preventing HIV infection (Table 1). By far, the most frequent primary concern of participants about getting circumcised was pain (63%); infection was the primary concern of 20%, and “sores on the penis” (injuries to the penis) was a primary concern of 10%. *“Not being part of my culture”* or *“Not part of my religion”* were mentioned by only one participant each. When asked whether their father was circumcised, significantly more Papuans responded that their father was uncircumcised (76%) than did non-Papuans (22%), with about a quarter of participants (23%) responding that they do not know their father’s circumcision status.

Forty-two participants (45%) reported that they had ever had sex: 25% among 15–19 year-olds and 75% among those 20 years and older. Just one participant indicated that he had ever had sex with another man. The mean number of sex partners among those who were sexually active in the last six months was 1.28, with the median and mode being 1. Just ten (24%) of those who had ever had sex had ever used a condom, and 5 (50%) of the 10 used a condom the last time they had sex.

Six weeks post-circumcision, participants were asked a series of questions to assess their level of satisfaction with their circumcision experience and their overall opinions about male circumcision and risk behaviors (Table 2). Ninety of the 94 (96%) participants were followed up at six weeks. All participants were fully healed. All but one said that they were somewhat satisfied (20%) or very satisfied (78%) with their circumcision, and all but three (96%) said that if they were to do it again, they would get circumcised. Reasons given for having misgivings were the length of time for the procedure and pain during the healing process. When asked if, since being circumcised, they are more of a man, 13% responded that they had no opinion, while 87% responded “somewhat true” (19%) and “very true” (68%). All but one participant said that all men in Papua should be circumcised, and all but two said that if they had a son, they would get him circumcised.

**Table 1:** Characteristics, beliefs, and reported behaviors of 94 males before being circumcised in three community health facilities in Nabire district, Papua, Indonesia

Variable	Total *N* (%)	Ethnicity			*p*-value
		Non-Papuan *N* (%)	Papuan *N* (%)	Test statistic	
Ethnic group you most identify with	94 (100)	36 (38.3)	58 (61.7)	χ^2^ = 5.15	0.02
Age mean (sd)	21.7 (8.8)	21.4 (8.1)	21.9 (9.3)	t = −0.26	0.80
Education (mean years)	12.3	11.42	12.9	t = −0.72	0.47
*Are you in school*				χ^2^ = 0.69	0.71
Yes	66 (70.2)	25 (69.4)	41 (70.7)		
No	27 (28.7	11 (30.6)	16 (27.6)		
No answer	1 (1.0)	0 (0)	1 (2)		
*Main reason you want circumcision*				χ^2^ = 1.84	0.77
Hygiene	71 (75.5)	27 (75)	44 (75.9)		
Family decision	9 (9.6)	3 (8.3)	6 (10.3)		
Reduce risk of HIV infection	11 (11.7)	4 (11.1)	7 (12.1)		
Religion	1 (1.1)	1 (2.8)	0 (0)		
Other	2 (2.1)	1 (2.8)	1 (1.7)		
*Main concern about getting circumcised*				χ^2^ = 4.27	0.64
Infection	19 (20.2)	6 (16.7)	13 (22.4)		
Pain	59 (62.8)	26 (72.2)	33 (56.9)		
Sores/wounds	9 (9.6)	2 (5.6)	7 (12.1)		
Against my culture	1 (1.1)	0 (0)	1 (1.7)		
Against my religion	1 (1.1)	0 (0)	1 (1.7)		
Other	5 (5.3)	2 (5.6)	3 (3.2)		
*Father circumcised?*				χ^2^ = 26.57	< 0.01
Circumcised	22 (23.4)	16 (44.4)	6 (10.3)		
Uncircumcised	52 (55.3)	8 (22.2)	44 (75.9)		
Don’t know	20 (21.3)	12 (33.3)	8 (13.8)		
*Have you ever had sex?*				χ^2^ = 1.76	0.41
Yes	42 (44.7)	13 (36.1)	29 (50)		
No	50 (53.2)	22 (21.1)	28 (48.3)		
Refuse to answer	2 (2.1)	1 (2.8)	1 (1.7)		
*Number of sex partners last 6 months* (*N* = 42)				χ^2^ = 2.11	0.55
0	13 (31.0)	3 (23.1)	10 (34.5)		
1	23 (54.8)	8 (61.5)	15 (51.7)		
2	4 (9.5)	2 (15.4)	2 (6.9)		
3 or more	2 (2.7)	0 (0)	2 (6.9)		
*Have you ever used a condom?* (*N* = 42)				χ^2^ = 0.22	0.64
Yes	10 (23.8)	2 (15.4)	8 (27.6)		
No	32 (76.2)	11 (84.6)	21 (72.4)		
*Did you use a condom last time you had sex?* (*N* = 10)				χ^2^ = 0	1.0
Yes	5 (50.0)	1 (50.0)	4 (50.0)		
No	5 (50.0)	1 (50.0)	4 (50.0)		

**Table 2:** Levels of satisfaction and reported sexual behaviors among 90 participants six weeks following circumcision in Nabire district, Papua, Indonesia

Variable	Total *N* (%)	Ethnicity			*p*-value
		Non-Papuan *N* (%)	Papuan *N* (%)	Test statistic	
*How satisfied are you with your circumcision?*				χ^2^ = 5.07	0.17
Very satisfied	70 (77.8)	31 (86.1)	39 (72.2)		
Somewhat satisfied	18 (20)	4 (11.1)	14 (25.9)		
Somewhat dissatisfied	1 (1.1)	1 (2.8)	0 (0)		
Very dissatisfied	0 (0)	0 (0)	0 (0)		
Don’t know/no answer	1 (1)	0 (0)	1 (1.8)		
*If I were to do it again, I would*				χ^2^ = 1.56	0.46
Get circumcised	86 (95.6)	34 (94.4)	52 (96.3)		
Remain uncircumcised	3 (3.3)	1 (2.8)	2 (3.7)		
Don’t know/no answer	1 (1.1)	1 (2.8)	0 (0)		
*Since I was circumcised, I am more of a man*				χ^2^ = 5.21	0.07
Very true	61 (67.8)	29 (80.6)	32 (59.3)		
Somewhat true	17 (18.9)	3 (8.3)	14 (26.0)		
Not very true	0 (0)	0 (0)	0 (0)		
Not at all true	0 (0)	0 (0)	0 (0)		
Don’t know/no answer	12 (13.3)	4 (11.1)	8 (14.8)		
*If I had my way, all men in Papua would be circumcised*				χ^2^ = 5.15	0.07
Strongly agree	76 (84.4)	33 (91.7)	43 (79.6)		
Somewhat agree	13 (14.4)	2 (5.6)	11 (20.4)		
Somewhat disagree	0 (0)	0 (0)	0 (0)		
Strongly disagree	0 (0)	0 (0)	0 (0)		
Don’t know/no answer	1 (1.1)	1 (2.8)	0 (0)		
*If I had a son, I would get him circumcised*				χ^2^ = 3.42	0.18
Strongly agree	78 (86.7)	34 (94.4)	44 (81.5)		
Somewhat agree	10 (11.1)	2 (5.6)	8 (14.8)		
Somewhat disagree	0 (0)	0 (0)	0 (0)		
Strongly disagree	0 (0)	0 (0)	0 (0)		
Don’t know/no answer	2 (2.2)	0 (0)	2 (3.7)		
*Have you had sex with a partner since circumcision*				χ^2^ = 4.95	0.08
Yes	7 (7.8)	2 (5.6)	5 (9.6)		
No	73 (81.1)	33 (91.7)	40 (74.1)		
Don’t know/no answer	10 (1.1)	1 (2.8)	9 (16.7)		
How many days after circumcision did you have sex? (*N* = 7) mean (SD)[min, max]	61 (26) [40,88]	64 (34) [40,88]	59 (26) [40,88]	t = 0.20	0.85
*The last time you had sex did you use a condom *(*N* = 7)				χ^2^ = 0	1.0
Yes	1 (14.3)	0 (0)	1 (20.0)		
No	6 (85.7)	2 (100)	4 (80.0)		

Notably, when asked the best age for circumcision, responses ranged from 3 years of age to 20 years, with 76 (84%) of respondents citing age 15 or below.

Participants were requested not to have sex in the six weeks after circumcision. Seven participants (8%), all of whom were over age 19 years, said that they had sex prior to the six-week visit; 10 refused to answer the question. All but one of those who had had sex said that it was with their spouse or live-in partner, and only one of the seven had used a condom with sex.

Participants were asked a series of questions to assess their levels of knowledge about circumcision, how they viewed themselves now that they were circumcised, and whether they might change their risk behaviors in view of their new circumcision status (Table 3). All but three participants (97%) said that they think people in their community view them differently now that they are circumcised. Seventy-eight respondents (87%) believed that it is very true or somewhat true that they are now more of a man since being circumcised. When asked, *“Since you were circumcised*,* do girls/women think you are more attractive?”* the great majority (82%) said that they didn’t know, while 14% said that it was very true or somewhat true. More Papuans (76%) than non-Papuans (58%) said that it was “not at all true” that they are now less Papuan since being circumcised; only 9% felt that the statement was true. Fifty-four participants (60%) responded that circumcised men are less likely to get infected with HIV compared to uncircumcised men, whereas 30 (33%) said they were more likely, and six (7%) said they did not know.

**Table 3:** Beliefs about male circumcision among 90 study participants six weeks following circumcision in Nabire district, Papua, Indonesia

Variable	Total *N* (%)	Ethnicity			*p*-value
		Non-Papuan *N* (%)	Papuan *N* (%)	Test statistic	
*Do people in your community view you differently now that you are circumcised?*				χ^2^ = 0	1.0
Yes	87 (96.7)	35 (97.2)	52 (96.3)		
No	0 (0)	0 (0)	0 (0)		
Don’t know/no answer	3 (3.3)	1 (2.8)	2 (3.7)		
*Since I was circumcised, I am more of a man*				χ^2^ = 5.21	0.07
Very true	61 (67.8)	29 (80.6)	32 (59.3)		
Somewhat true	17 (18.9)	3 (8.3)	14 (26.0)		
Not very true	0 (0)	0 (0)	0 (0)		
Not at all true	0 (0)	0 (0)	0 (0)		
Don’t know/no answer	12 (13.3)	4 (11.1)	8 (14.8)		
*Since I was circumcised girls/women think I am more attractive*				χ^2^ = 1.03	0.79
Strongly agree	5 (5.6)	1 (2.8)	4 (7.4)		
Somewhat agree	7 (7.8)	3(8.3)	4 (7.4)		
Somewhat disagree	0 (0)	0(0)	0 (0)		
Strongly disagree	4 (4.4)	2 (5.6)	2 (3.7)		
Don’t know/no answer	74 (82.2)	30 (83.3)	44 (81.5)		
*Since I was circumcised, I am less Papuan*				χ^2^ = 7.45	0.02
Strongly agree	6 (6.7)	1 (2.8)	5 (9.3)		
Somewhat agree	0 (0)	0	0		
Somewhat disagree	0 (0)	0	0		
Strongly disagree	62(68.9)	21 (58.3)	41 (75.9)		
Don’t know/no answer	22(24.4)	14 (38.9)	8 (14.8)		
*What is the best age to circumcise (yrs)*				χ^2^ = 1.89	0.60
1–9	6 (6.7)	2 (5.6)	4 (7.4)		
10–14	37 (41.1)	14 (38.9)	23 (42.6)		
15–19	45 (50.0)	20 (55.6)	25 (46.3)		
20+	2 (2.2)	0 (0.0)	2 (3.7)		

When asked about whether circumcised or uncircumcised men get more sexual pleasure, 45 participants (50%) believed that circumcised men get more pleasure, 36 (40%) said that they didn’t know, and 8 participants (9%) said each gets about the same. Similarly, 40 participants (44%) believed that the female partners of circumcised men experience more sexual pleasure than those of uncircumcised men, with 42 (47%) saying that they didn’t know if there was any difference and 7 (8%) saying about the same.

Participants were asked an open-ended question, *“What are some of the things that could be done to improve the VMMC services that you received?”* Suggestions included: reduce waiting time, reduce the time of the procedure, and make the injection less painful. The large majority said that the services should be continued and should be promoted to youth.

## Discussion

HIV prevalence in Papua Indonesia is high—nearly ten times higher than in the rest of Indonesia—and, unlike the rest of Indonesia, in Papua almost all new HIV infections occur through heterosexual transmission [3]. Further, while Indonesia is a predominantly Muslim country and male circumcision is nearly universal, in Papua it is rarely practiced [5]. This combination of characteristics—relatively high HIV prevalence, predominantly heterosexual HIV transmission, low prevalence of male circumcision—suggest that VMMC could have a significant impact on the HIV epidemic in Papua if safe, affordable VMMC services could be widely available and uptake by Papuans were substantial [11]. In recognition of this, the Republic of Indonesia’s Ministry of Health, along with major international agencies, has endorsed its use [5, 11].

We sought to develop and pilot a Papuan indigenous model for VMMC through extensive consultation with local community members and in close collaboration with the District Hospital authorities in Nabire District, which has among the highest HIV prevalence in Papua. While we found strong support for the introduction of comprehensive VMMC services for HIV prevention among government officials, community leaders, teachers, students and community health providers, after doing the training and building the capacity for safe services at three community health centers, uptake by adolescent and adult Papuan men was poorer than expected. Before onset of project activities, we projected demand for 400 circumcisions in a four-month period. We considered that target as conservative, given our experience in African communities where VMMC is not traditionally practiced. In our experience, initial pent-up demand makes the first few hundred circumcisions not difficult to achieve. Also, during our community engagement, the target of 400 seemed very achievable according to the providers, elders, parents, and teachers we consulted. Yet despite numerous informational sessions and widespread demand creation efforts conducted with peers and community members, just 104 young and adult men volunteered for VMMC, and of these, 94 qualified for and accepted circumcision. The one previous initiative to introduce VMMC was a clinic-based pilot project conducted in 2012–2015 jointly by the Indonesian MOH, the Australian government (AUSAID), and the Clinton Health Access Initiative using the PrePex device. The study consisted of a situational analysis followed by a pilot implementation of VMMC in 4 cities [4]. Consistent with our findings, acceptance was poor. A total of 411 men received PrePex device placements out of a targeted 800. An assessment of the initiative concluded that the overall demand for circumcision in Tanah Papua was weak.

Despite discouraging results from the previous and this attempt to promote uptake of VMMC services, our study did establish very high levels of satisfaction among the adolescents and men who did volunteer for circumcision, with nearly all participants saying that they would do it again if given the choice, that they would recommend it to others and that VMMC services should continue to be available. In addition, our study showed that high levels of safety could be achieved in community health settings with mostly nurses performing the surgery with no serious and just two moderate adverse events—levels of adverse events comparable to the 1.7–3.6% rates achieved during the three randomized controlled trials of VMMC performed in three countries in sub-Saharan Africa [7–9].

Surprisingly, participants cited neither religion nor culture as significant factors influencing their MC decision. Possibly, those who saw religion or culture as barriers to VMMC did not volunteer for the study. Participant responses contrasted markedly with concerns voiced during meetings and focus groups where community members inevitably mentioned that circumcision is not part of traditional Papuan culture, and many indigenous Papuans may see the promotion of circumcision as an attempt by outsiders to change Papuan culture to become more like other Indonesian cultures. Similarly, concerns were expressed that by circumcising their sons, some families could be perceived by others as converting to Islam, which could be stigmatizing. Similar cultural and religious concerns arose when VMMC programs were first introduced in many African communities. It required concerted efforts and, in some cases, several years to allay concerns and achieve significant demand for VMMC [12–14]. As in those African settings, health providers and community leaders in Nabire emphasized the hygiene, health, and disease prevention aspects of VMMC to assure youth and parents that the promotion of VMMC was not an attempt by non-Papuans to impose their cultural values or differing religious customs on Papuans. While Papuans appeared very receptive to such messaging, the poor uptake of VMMC services was likely in significant part attributable to these two salient concerns. As in many African communities, it will require additional resources and time to address all the concerns of Papuans regarding the benefits of VMMC and how it can be integrated into the values and norms of the local cultures and religions.

Notably, most of those who volunteered to be circumcised stated that the main reason for seeking circumcision was for reasons of hygiene (76%); whereas to reduce risk of HIV infection was chosen by just 12% of participants. Perhaps in keeping with these responses, just 60% of participants were able to answer the question correctly whether circumcised or uncircumcised men are at less risk of HIV infection. During our community outreach and education events and in our written dissemination materials, we emphasized the 60% protective effect of VMMC. In addition, during the consent process, counselors made sure that volunteers fully understood both the risks and benefits of VMMC. However, because HIV is still very stigmatized in Papua, during our discussions with local stakeholders and community members at both the provincial and district levels, concern was expressed that if HIV is emphasized, Papuans may feel that they/their sons are being accused of having “free” sex. Consequently, they urged emphasis on improved hygiene as a salient message regarding the benefit of circumcision. The messages regarding improved hygiene seem to have fallen on more receptive ears or were delivered more forcefully than those regarding the reduction in HIV risk. Further study is necessary to learn how better to fully inform community members and VMMC volunteers regarding the preventive benefits of VMMC.

In this study we offered VMMC services to any males 15 years and above, but we focused our demand creation efforts on young men ages 15–19 years and their parents. Nevertheless, just 53% of males circumcised were in that age range. While these results suggest that there is demand for VMMC among men in their twenties and thirties, reports from our community meetings and from parents seeking VMMC for their sons at the health facilities indicate that substantial demand is in the age range of 8 to 15 years and very few would prefer circumcision above age 20 years. When asked their opinion as to the best age to be circumcised, nearly half (47.8%) of our participants expressed a preference for below 15 years, and just two men (2.2%) felt that over age 20 years would be preferable. Experience from multiple African countries indicates that adolescents accept VMMC more readily than their older counterparts [15]. Despite herculean efforts and millions of dollars allocated to create demand for VMMC among men in their twenties and older, VMMC programs in Africa have had the greatest success attracting adolescents below the age of 18 years for circumcision [16]. While several years of delay occur before the greatest number of infections are averted, adolescent VMMC ultimately has the greatest impact on the epidemic due to the much greater proportions of males circumcised as they enter the highest HIV incidence years [17]. We originally proposed to focus demand creation efforts on boys ages 10–16 years. However, in keeping with WHO and The U.S. President’s Emergency Plan for AIDS Relief (PEPFAR) guidelines [18], circumcision for this age group would require the use of the Shang Ring. In attempting to adopt this method, we found that government agencies and local providers did not want to introduce it into the country for reasons of cost and the need for a regular supply chain in the future. Future attempts to introduce VMMC in Papua should reassess possibilities for offering VMMC services to boys below 15 years of age using this procedure.

In other settings, the most efficient means of accessing and providing VMMC services to large numbers of young males occurs at primary and secondary schools where teachers, parents and students are eager for HIV prevention information and services [19–21]. Delivering circumcision information to adolescents through school-based programs offers an efficient means to reach large numbers of male youth, along with providing easy logistical access to VMMC and post-procedural medical monitoring [22]. In Papua, however, students often attend schools far from their resident villages, making contact with parents, who must be part of the MC decision, extremely difficult. A major challenge to the uptake of VMMC among school-attending adolescents is reaching parents to discuss the benefits and risks of VMMC and to obtain their informed consent for the procedure. Several different approaches to overcoming these challenges have been considered, including deploying mobile teams, direct telephone communications, and concentrating VMMC services at times when parents visit schools. All such proposed solutions, however, proved logistically challenging, costly, or inefficient.

There were several limitations to this research. Political disruptions and religious holidays delayed and interrupted the research program, as did the COVID epidemic. Had time and funding been less limited, educating more community members and building more support for the VMMC program may have been possible. This remains a possibility, although numerous other challenges were also present (see below). We were unable to assess acceptability as originally proposed (i.e., the proportion of those exposed to VMMC messages who volunteered for VMMC) due to our inability to fully record the individuals who experienced our educational and outreach activities. The study was limited to just one region of Papua around the western coastal city of Nabire. The results may not be generalizable to all of Papua. For example, the diverse communities in the interior highlands and those on the southern coast may differ in their attitudes and beliefs about HIV risk and male circumcision.

Based on our study results and the lessons learned during our extensive efforts working with community stakeholders to create a truly Papuan model for the introduction of VMMC services in the region, we are skeptical whether VMMC can be a cost-effective means of reducing HIV incidence in Tanah Papua. Great diversity in cultural and religious traditions across numerous different communities, low population densities with large distances and difficult communications between communities, low levels of knowledge about HIV, the lack of infrastructure and resources, especially compared to other regions in Indonesia, and the moderate HIV incidence compared to many regions of sub-Saharan Africa where VMMC has been introduced—all these factors and more converge to make uptake and successful scale-up of VMMC more challenging than any other global region where VMMC has been implemented.

It is unlikely that VMMC can be made cost-effective in the Papuan setting—the cost per new HIV infection averted would be high due to the moderate HIV incidence, the logistical challenges, and the large amount of resources required to achieve each circumcision. The introduction of currently unavailable pre-exposure prophylaxis (PrEP), especially the newly developing longer-acting forms of PrEP [23], could be a more efficient means of HIV prevention in Papua for both men and women. Nevertheless, VMMC, because it is a proven HIV prevention strategy, should be made available for those who wish it. During our dissemination events that included religious leaders, community members, stakeholders in the Education and Health Offices, and providers in the community health centers, there was broad consensus that a VMMC program should be supported. The option of safe, affordable VMMC should be made available at a minimum of selected health facilities. This would entail greater resource investment than currently available from local, national and international stakeholders and should include information about the benefits and risks of VMMC integrated into existing health facility-based outreach programs and school activities.
